# Unmet Need for Family Planning in Nepal during the First Two Years Postpartum

**DOI:** 10.1155/2014/649567

**Published:** 2014-06-05

**Authors:** Suresh Mehata, Yuba Raj Paudel, Ranju Mehta, Maureen Dariang, Pradeep Poudel, Sarah Barnett

**Affiliations:** ^1^Nepal Health Sector Support Programme, Ministry of Health and Population, Ramshah Path, Kathmandu 44600, Nepal; ^2^Karuna Foundation Nepal, Baluwatar, Kathmandu 44616, Nepal; ^3^Kist Medical College, Imadol, Lalitpur 44705, Nepal; ^4^Options Consultancy Services Limited, Devon House, 58 St Katharine's Way, London E1W 1LB, UK

## Abstract

Contraceptive use during the postpartum period is critical for maternal and child health. However, little is known about the use of family planning and the determinants in Nepal during this period. This study explored pregnancy spacing, unmet need, family planning use, and fertility behaviour among postpartum women in Nepal using child level data from the Nepal Demographic and Health Surveys 2011. More than one-quarter of women who gave birth in the last five years became pregnant within 24 months of giving birth and 52% had an unmet need for family planning within 24 months postpartum. Significantly higher rates of unmet need were found among rural and hill residents, the poorest quintile, and Muslims. Despite wanting to space or limit pregnancies, nonuse of modern family planning methods by women and returned fertility increased the risk of unintended pregnancy. High unmet need for family planning in Nepal, especially in high risk groups, indicates the need for more equitable and higher quality postpartum family planning services, including availability of range of methods and counselling which will help to further reduce maternal, perinatal, and neonatal morbidity and mortality in Nepal.

## 1. Background

For better maternal and child health outcomes, an interval of at least 24 months following birth is recommended before becoming pregnant again. Evidence suggests that family planning can avert more than 30% of maternal and 10% of child mortality if pregnancies are spaced more than 24 months apart [1]. However, interpregnancy intervals are frequently shorter due to poor awareness of the risks associated with short pregnancy intervals among both women and health workers, resulting in the low uptake of contraceptives during this period [1, 2]. One study based on Demographic Health Survey (DHS) data from 25 countries revealed that one-fifth of postpartum women who had resumed menstruation and were not abstaining from sexual intercourse were not using contraception, and among these women, two-thirds wanted to either space or limit their childbearing [3].

Postpartum family planning (PPFP) plays a vital role in preventing unintended pregnancies and reducing maternal and child mortality [4]. It promotes the health of mothers and children by lengthening pregnancy interval and helps to avoid financial, psychological, and health costs due to unintended pregnancies. However, following childbirth many families overlook contraception due to a poor perception of pregnancy risks, difficulty in accessing services, and sociocultural issues [5, 6]. Many factors such as geographical and financial access, provider bias, poor method choice, lower status of women, medicolegal restrictions, and fear of side effects act as a barrier to family planning use [7].

The Government of Nepal (GoN), Family Health Division (FHD), has been strengthening family planning counselling and increasing the availability of family planning methods. The Nepal Demographic and Health Survey (NDHS) 2011 showed that only 9% of women who had a live birth in the five years preceding the survey were given information or counselled on family planning during a postpartum checkup [8]. This suggests there are missed opportunities to provide information and counselling on family planning methods and services to postpartum women. Studies conducted in Nepal have shown that unintended pregnancies, due to low use of contraception and reliance on less effective family planning methods (traditional methods), have caused repeated abortions and abortion-related complications [9, 10]. Therefore, to consolidate and extend the achievements made in maternal and child health, increased access to quality family planning services and strengthened postpartum counselling will be instrumental. Uptake of postpartum family planning has been underinvestigated in Nepal. This paper looks at the interval between births and subsequent pregnancies, the risk of fertility return, and the level and determinants of unmet need in Nepal during the first two years postpartum using the data from NDHS 2011.

## 2. Methods

### 2.1. Data Source and Sampling Strategy

This study utilized data from the NDHS 2011, and full details of the NDHS methodology, sampling procedure, and questionnaires are available in the report [8]. In brief, the NDHS 2011 used two-stage stratified cluster sampling to select a representative sample of households. The primary objective was to provide national estimates with an acceptable level of precision for population characteristics such as fertility, contraceptive need and prevalence, and selected health indicators and infant mortality. A total of 11,353 households were selected from 289 primary sampling units (194 rural and 95 urban) using probability proportionate to size. A response rate of 99% was reported for occupied households. The survey successfully completed 5391 interviews with women who had a child in the last five years, and 2030 of these had given birth in last 24 months.

### 2.2. Variables Used for Analysis

The dependent variable in this analysis was unmet need for family planning. It was obtained prospectively as it is more likely to correlate with the need for family planning during the postpartum period (taken as the first two years postpartum for this analysis) [11]. The indicators analysed in this study are defined in Table 1. Factors related to fertility returns considered in this analysis were resumption of menses, initiation of sexually activity, and nonexclusive breast feeding.

Three major predictor variables were included: maternal factors (such as age and education), household-level variables (such as caste/ethnic group and wealth quintile), and community-level variables (such as urban/rural residence and ecological zone). The method used to compute the wealth index is described in the NHDS 2011 report [8].

### 2.3. Data Analysis

All analyses were performed using the national sample using SPSS 16. Reported values were weighted by sample weights to provide population estimates. The Chi-square test was used to measure the association between the factors and outcome variables using complex survey design, considering clusters, and stratification by urban/rural location. A *P* < 0.05 was considered to be statistically significant. Significant variables were analysed using survey logistic regression methods.

## 3. Results

**Figure 1:**
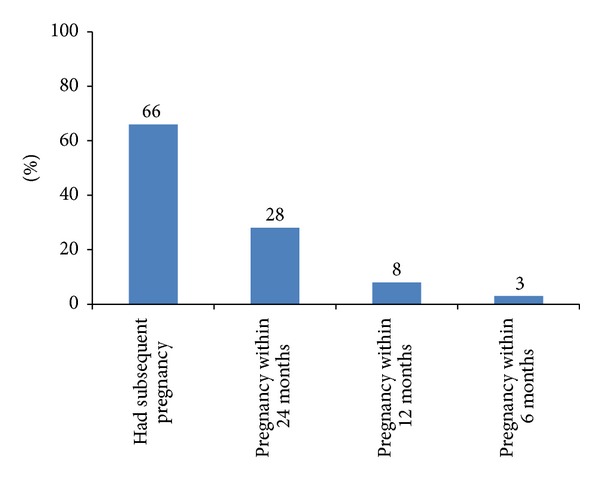
Subsequent pregnancy, and interval between birth and subsequent pregnancy, among women aged 15–49 who gave birth in the last five years (*N* = 5391).

Among the 5391 surveyed women who gave birth in the last five years, 66% had a subsequent pregnancy, with 28% becoming pregnant within 24 months of their previous birth, 8% within 12 months, and 3% within six months (Figure 1).

Younger women who became pregnant were more likely to have a shorter interval between birth and a subsequent pregnancy than older women (Table 2). One-third (33%) of those under 20 years of age at the first birth had a pregnancy interval of less than 24 months, compared to 23% of those aged 20–29 at their first birth and 14% of those who were at least 30. The lower the level of education the higher the likelihood of having a shorter interval: 36% of women with no education had an interval of less than 24 months compared to 11% of those with higher education. Furthermore, the lower the socioeconomic status the smaller the interval: almost two-fifths (39%) of women in the poorest quintile had an interval of less than 24 months compared to 17% of those in the highest quintile. There were substantial differences between caste/ethnic groups in the length of the interval, ranging from 16% of Newars having an interval of less than 24 months compared to 45% of Muslims. Those residing in rural areas (29%) were more likely to have an interval of less than 24 months, than those residing in urban areas (21%). There were no significant differences in the length of the interval by ecological zone.

**Figure 2:**
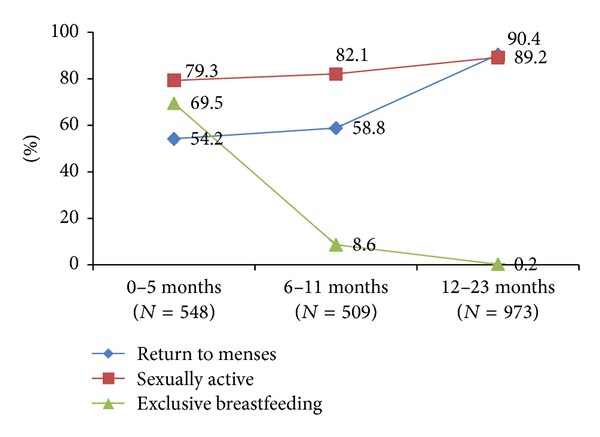
Pregnancy risk at 0–23 months postpartum.

**Figure 3:**
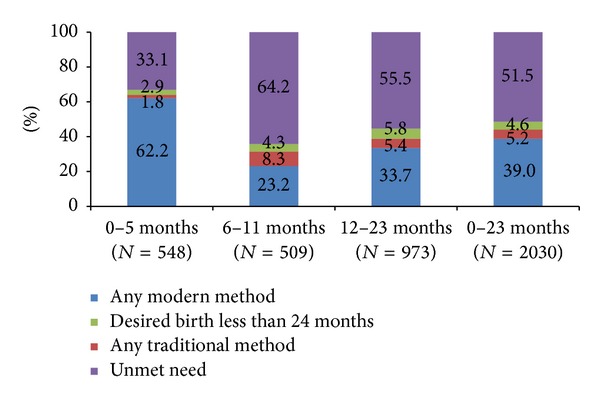
Uptake of family planning among women 0–23 months postpartum.

Figure 2 shows that between 12 and 23 months postpartum, most women were sexually active (89%) and menstruating (90%). However, Figure 3 shows that, although only 5% of women 0–23 months postpartum stipulated that they wanted to have another child within two years, only 44% were using any method of family planning and only 39% were using a modern method. A further 5% were reliant on traditional family planning methods, leaving an unmet need among more than half (52%) of women at 0–23 months postpartum. The uptake of a modern method was highest among women who were 0–5 months postpartum (63%), largely due to the attribution of the LAM (57%). Less than a quarter of women were using modern methods at 6–11 months postpartum (24%), and this rose slowly to just over one-third (34%) at 12–23 months postpartum.

**Figure 4:**
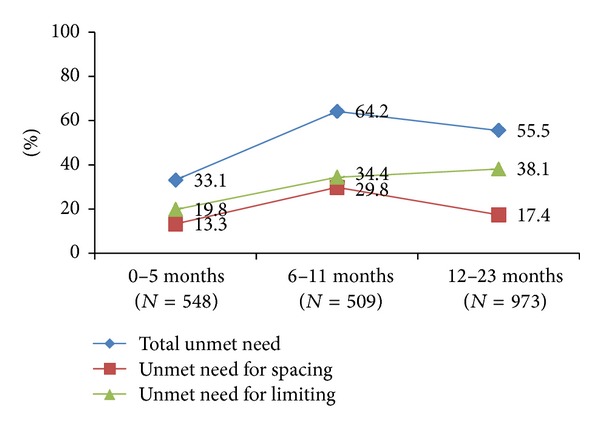
Unmet need at 0–23 months postpartum.

More than half of women (52%) who had delivered within the past 24 months had unmet need for family planning. Unmet need for limiting is slightly higher than unmet need for spacing up to 12 months postpartum, and the difference becomes more substantial at 12–23 months (38% have an unmet need for limiting and 17% have an unmet need for spacing) (Figure 4).

Table 3 shows the factors associated with unmet need (spacing, limiting, and total) using the Chi-square test. This analysis indicates that women who were over the age of 29, have no education, are in the poorest quintile, reside in rural and hill areas, and are Muslim had a higher total unmet need for family planning compared to women who were below 20 years, had a higher education, are in the richest quintile, reside in urban and Terai areas, and are Newars.

Younger women aged <20 years had more than five times higher unmet need for spacing (32%) than women aged over 29 years (6%). Other groups with higher levels of unmet need for spacing were Terai caste women (29%), women with secondary level education (22%), women from the richer quintile (20%), and women living in rural (20%) and mountain areas (18%).

Women aged above 29 years (50%), in the poorest quintile (40%), living in hill (39%) or rural areas (33%), with no formal education (37%), and being Muslims (48%) had a higher unmet need for limiting.

Table 4 shows the multinomial logistics regression analysis for the determinants of unmet need among women 0–23 months postpartum. Rural residents [adjusted odds ratio (aOR) 1.57; 95% CI: 1.07, 2.30] and Muslims (aOR 2.47; 95% CI: 1.51, 4.05) had higher odds of unmet need when compared with urban and Brahmin/Chettri women, respectively.

Younger women had a greater unmet need for spacing: the odds of unmet need for spacing were lower among women over 29 years (aOR 0.12; 95% CI: 0.10, 0.28) and for those between 20 and 29 years (aOR 0.63; 95% CI: 0.43, 0.92), compared to women aged less than 20 years. Wealthier women had lower unmet need: the odds were lower among the richest quintile (aOR 0.30; 95% CI: 0.15, 0.58) compared to the poorest quintile. However, higher odds were noted among secondary level educated women when compared to those with no education (aOR 1.62; 95% CI: 1.10, 2.37). Higher odds of unmet need for spacing were seen among the other Terai caste (aOR 1.91; 95% CI: 1.07, 3.41) compared to Brahmin/Chhetri.

In contrast to spacing, older women had greater unmet need for limiting: the odds of unmet need for limiting were higher among women over 29 years (aOR 4.25; 95% CI: 2.51, 7.17) and among women aged 20–29 years (aOR 1.89; 95% CI: 1.18, 3.01) compared to those under 20. Rural residents (aOR 1.83; 95% CI: 1.21, 2.75) had greater unmet need for limiting compared to urban residents; and Muslims (aOR 2.58; 95% CI: 1.47, 4.57) had greater unmet need for limiting compared to Brahmin/Chhetri. The odds were lower among women with secondary education (aOR 0.71; 95% CI: 0.51, 0.99) compared to those with no education and women residing in the Terai (aOR 0.64; 95% CI: 0.42, 0.97) compared to mountain districts.

## 4. Discussion

### 4.1. Birth to Pregnancy Intervals and Determinants

The recommended interval between a birth and a subsequent pregnancy is at least twenty-four months [1]. Pregnancy intervals of six months or less are associated with an elevated risk of maternal mortality; and an interval of eighteen months or less is associated with an elevated risk of infant, neonatal, and perinatal mortality, low birth weight, small size for gestational age, and preterm delivery [12, 13]. Further analysis of DHS surveys in Nepal from 2001 to 2011 showed an association between neonatal mortality and a short birth interval (under two years) [14]. A meta-analysis also revealed that appropriate spacing of pregnancies could help prevent adverse perinatal outcomes [15]. This study has shown that more than a quarter of women who gave birth in the last five years became pregnant within 24 months of their previous birth. Other Asian and African countries have also reported high percentages of pregnancies occurring within 24 months: Uttar Pradesh, India (30%) [16], Pakistan (60%) [17], Liberia (43%) [18], and Kenya (50%) [19]. This study showed that women who gave birth at less than 20 years of age were more likely to have a subsequent pregnancy within 24 months. Similarly, rural residence, lack of education, being in the poorest quintile, and being Muslim were associated with a pregnancy interval of less than 24 months. The median duration of the birth to pregnancy interval in Nepal is 24 months, similar to a Nigerian study among women attending antenatal and FP clinics that showed a median interbirth interval of 21.5 months [20]. There is a need to increase the promotion of adequate spacing between pregnancies, especially among the higher risk groups (age at first birth below 20, no education, poorest quintiles, Muslims, and rural women). A previously conducted randomized control trial in Nepal demonstrated the effectiveness of individual health education on postpartum family planning uptake, which suggests that health education could be a vehicle for promotion of healthy timing and spacing [21].

### 4.2. Fertility Return and Risk of Pregnancy

The return of fertility is often unpredictable among postpartum women [22]. Most nonlactating women will begin to ovulate at around six weeks postpartum [23]. However, breastfeeding delays the resumption of ovulation and can be a reliable form of contraception within the first six months if menses have not returned and the mother is exclusively breastfeeding [23]. The longer the duration between birth and resumption of menses is, the more likely the ovulation will precede the return to menses [24]. Periods of abstinence from sexual activity after a birth vary largely. One study has indicated that, among those practicing postpartum abstinence, sexual activity may initially be irregular, progressing to regular activity later [25]. This study has shown that even though large numbers of couples were sexually active (80%) and menses had returned (54%) during the first six months postpartum, the use of family planning during the postpartum period was often neglected. A similar pattern has been seen in Bangladesh, where 90% women resumed sexual activity from three to six months postpartum [11]. This indicates the need for effective counselling to space or prevent unintended pregnancies. A previous study from Nepal showed poor awareness of the timing of fertility return [26], and many health workers overlook family planning counselling during antenatal, postpartum, and infant health care [27]. Furthermore, the benefits of family planning go beyond improvements in maternal and child health, as use of family planning can result in higher educational attainment, better employment opportunities, higher socioeconomic status, and female empowerment [28].

### 4.3. Family Planning Need and Uptake

Low uptake of the family planning methods or high unmet need for family planning is the major cause of unintended pregnancies and short pregnancy intervals in developing countries [29]. This study revealed a far higher unmet need for family planning among postpartum women (52%), compared to all married women of reproductive age (MWRA) (27%), suggesting that postpartum women should be a priority target group for family planning programmes [8]. The higher unmet need for limiting than for spacing among postpartum women and MWRA (17% for limiting and 10% for spacing) [8] suggests a need for a review of overall family planning efforts, for instance, increasing the availability of contraceptive method mix for women during postpartum/infant care, including long acting and permanent methods [30].

With the increase in institutional deliveries in Nepal [8] there is an opportunity to reach more women through predischarge information, counselling, and services including postpartum intrauterine copper device (PPIUCD). In addition, routine immunization coverage in Nepal is high (almost 90% through facility based or community based services) [8]; thus there is potential for integration of the Expanded Programme of Immunisation (EPI) and PPFP, which may help improve the delivery of family planning information, counselling, and referrals. Additionally, the promotion of LAM should be a programmatic approach, as more than two-thirds of women are breastfeeding exclusively up to six months, indicating the potential for integrating information on EBF, LAM, and pregnancy timing and spacing within the EPI and other community based safe motherhood programmes. Information, counselling, and referral for transition from LAM to other PPFP methods could also be promoted through EPI clinics [30].

### 4.4. Unmet Need for Family Planning in Postpartum Mothers

Literature regarding the determinants of unmet need in postpartum contraception is very scarce; however, studies investigating the determinants of family planning use by MWRA suggest a range of health systems; demographic, socioeconomic, and contextual factors have an effect on contraceptive use. Multinomial logistic regression in the current study is consistent with other studies, showing the richest wealth quintile had significantly lower odds of unmet need for spacing in comparison to poorest wealth quintile [31]. Since stockouts of family planning methods are common in public health facilities in Nepal [32], poor women might not be able to afford to purchase them from private facilities and hence may experience unmet need. Most of the poor in Nepal live in rural areas, where private facilities are often lacking and even many public health facilities are geographically inaccessible, making it difficult for rural women to access family planning methods. Consistent with a previous study among MWRA, Muslims had significantly higher unmet need in comparison to Brahmins/Chhettris in Nepal [33].

Women residing in the Terai area had significantly lower odds of unmet need for limiting which suggests that postpartum women in the mountain and hill areas may have less access to permanent methods. Mountain areas (3.4) also have a higher total fertility rate (TFR) in comparison to hill (2.6) and Terai (2.5) area and a lower contraceptive prevalence rate [8].

### 4.5. Strengths and Limitations

NDHS data provides nationally representative findings; however, women who were not using contraceptives, but were abstaining from sexual intercourse, were not considered to be protected from unintended pregnancy; however, considering changing sexual patterns and sexual activity during postpartum period [11, 25], we believe that these groups can be considered to have an unmet need. The NDHS dataset did not enable an exploration of differences in the uptake of family planning between women who did and did not use maternal, newborn, and child health services or received advice/counselling on family planning around the time of the birth of the previous child. The NDHS is a household survey and does not include health service data; hence this study did not access the availability of family planning methods or counselling, provider competency, or quality of care. The study is limited to quantitative analysis, and some qualitative research would provide a full understanding of factors affecting uptake of family planning during the period.

### 4.6. Further Research Priorities

Qualitative research would help to better understand the barriers to postpartum family planning in Nepal. Additionally the impact of providing family planning services during maternal and child health services needs further evaluation.

## 5. Conclusions

Among women who gave birth in the last five years, more than a quarter became pregnant within 24 months of their previous birth, despite the increased risk to maternal and newborn health. Family planning programmes need to work in close coordination with safe motherhood and child health programmes, discussing reproductive intentions for spacing or limiting and ensuring equitable access to a range of family planning methods with pregnant women during ANC, PNC, and EPI. The presence of large inequalities suggests high risk groups need additional targeting.

## Figures and Tables

**Table 1:** List of indicators included in this study and their definitions.

Variables	Definition/measurement
Unmet need	All postpartum women who are not currently using any family planning method were considered to have an unmet need for family planning. Unmet need was measured based on the DHS question “Would you like your next child within the next two years or would you like no more children?”

Return to menses	Postpartum amenorrhoea is the interval between the birth of a child and the resumption of menses and was assessed by women answering “yes” to the question “Has your menstrual period returned since the birth of (name)?” who were further asked “For how many months after the birth of (name) did you not have a period?”

Sexual activity	Postpartum sexual activity was assessed through questions: have you had sexual intercourse since the birth of (child's name)? Women who answered “yes” were further asked “For how many months after the birth of (child's name) did you not have sexual intercourse?”

Exclusive breastfeeding	Exclusive breastfeeding was considered if women were breastfeeding and did not give any additional food or liquid (including water) to their baby in the last 24 hours, excluding vitamins, medicines, and vaccines.

Family planning use	Family planning use was further divided into modern and traditional methods use. And the modern family planning methods included female sterilisation, male sterilisation, pills, intrauterine contraceptive device (IUCD), injectables, implants, male condoms, female condoms, diaphragm, foam/jelly, and lactational amenorrhoea method (LAM). Traditional/folk methods included rhythm, withdrawal, and folk methods.

Lactational amenorrhoea method (LAM)	The criteria used for LAM were exclusive breastfeeding and menses not returned since delivery and the infant is less than six months old.

**Table 2:** Demographic and socioeconomic characteristics of women aged 15–49 who gave birth in the last five years, by interval to subsequent pregnancy.

	No subsequent pregnancy		Subsequent pregnancy				Gave birth in last five years (*N*)	*P*
			≥24 months after previous birth		<24 months after previous birth	
	*N*	%	*N*	%	*N*	%
Age at first birth (years)								
<20	828	28.1	1154	39.2	960	32.6	2941	<0.001
20–29	978	40.9	854	35.7	561	23.4	2393	
≥30	34	59.6	15	26.3	8	14.0	57	
Level of education								
No education	476	18.7	1148	45.0	926	36.3	2550	<0.001
Primary	382	35.4	385	35.7	312	29.0	1079	
Secondary	794	54.1	417	28.4	257	17.5	1468	
Higher	188	63.9	74	24.9	33	11.2	295	
Wealth quintile								
Poorest	291	20.9	555	39.9	544	39.1	1390	<0.001
Poorer	353	29.8	488	41.3	342	28.9	1182	
Middle	430	37.9	399	35.2	304	26.9	1133	
Richer	414	44.2	311	33.2	212	22.6	938	
Richest	352	47.0	270	36.1	126	16.9	748	
Caste/ethnicity*								
Brahmin/Chhetri	586	36.2	598	37.0	434	26.8	1618	<0.001
Other Terai caste	166	29.7	208	37.3	184	33.0	558	
Dalit	269	28.0	377	39.3	313	32.7	959	
Newar	57	40.3	62	43.6	23	16.0	142	
Janjati	658	37.6	677	38.7	415	23.7	1751	
Muslim	95	27.0	99	28.3	157	44.8	351	
Place of residence								
Urban	214	42.5	184	36.6	105	20.9	503	<0.001
Rural	1626	33.3	1839	37.6	1423	29.1	4888	
Ecological zone								
Mountain	117	27.3	164	38.3	147	34.4	428	0.143
Hill	706	33.2	807	37.9	617	29.0	2130	
Terai	1017	35.9	1052	37.1	764	27.0	2833	
Total	**1840**	**34.1**	**2023**	**37.6**	**1528**	**28.3**	**5391**	

*Missing = 13.

**Table 3:** Demographic and socioeconomic characteristics of women 0–23 months postpartum with unmet need for family planning.

	Unmet need						Total women in 0–23 months postpartum period (*N*)	*P*
	All		For spacing		For limiting	
	*N*	%	*N*	%	*N*	%
Age (years)								
<20	131	49.3	85	32.0	46	17.3	265	<0.001
20–29	686	50.6	283	20.8	403	29.7	1358	
≥30	231	57.0	26	6.4	205	50.4	407	
Level of education								
No education	472	54.7	156	18.1	316	36.6	862	0.049
Primary	205	52.3	75	19.1	130	33.2	392	
Secondary	305	48.2	142	22.4	163	25.8	632	
Higher	66	45.9	22	15.0	44	30.9	144	
Wealth quintile								
Poorest	288	58.7	91	18.5	197	40.2	489	<0.001
Poorer	213	49.9	73	17.1	140	32.8	428	
Middle	260	55.4	130	27.7	130	27.7	469	
Richer	174	47.1	72	19.5	102	27.6	370	
Richest	113	41.2	28	10.3	85	30.9	274	
Caste/ethnicity*								
Brahmin/Chhetri	298	50.5	94	15.9	204	34.6	591	0.007
Other Terai caste	117	52.7	65	29.3	52	23.4	222	
Dalit	188	51.7	85	23.3	103	28.4	364	
Newar	22	41.8	6	11.8	16	30.0	52	
Janjati	323	49.9	115	17.8	208	32.1	647	
Muslim	98	66.0	27	18.2	71	47.8	148	
Place of residence								
Urban	74	39.3	31	16.4	43	22.9	189	0.001
Rural	974	52.9	363	19.7	611	33.2	1841	
Ecological zone								
Mountain	87	52.2	30	17.8	57	34.4	166	0.001
Hill	441	56.1	135	17.2	306	38.9	785	
Terai	520	48.2	229	21.2	291	27.0	1079	
Total	**1048**	**51.5**	**394**	**19.4**	**654**	**32.2**	**2030**	

*Missing = 6.

**Table 4:** Determinants of unmet need for family planning 0–23 months postpartum.

	Unmet need		
	All	For spacing	For limiting
	Adjusted OR (95% CI)	Adjusted OR (95% CI)	Adjusted OR (95% CI)
Age (years)			
<20 (ref.)	1	1	1
20–29	1.05 (0.75, 1.48)	0.63 (0.43, 0.92)*	1.89 (1.18, 3.01)*
≥30	1.31 (0.88, 1.97)	0.12 (0.10, 0.28)*	4.25 (2.51, 7.17)*
Level of education			
No education (ref.)	1	1	1
Primary	1.05 (0.76, 1.45)	1.12 (0.70, 1.78)	0.99 (0.70, 1.40)
Secondary	1.00 (0.75, 1.34)	1.62 (1.10, 2.37)*	0.71 (0.51, 0.99)*
Higher	1.02 (0.59, 1.81)	1.86 (0.97, 3.56)	0.69 (0.36, 1.29)
Wealth quintile			
Poorest (ref.)	1	1	1
Poorer	0.74 (0.55, 1.01)	0.70 (0.44, 1.10)	0.93 (0.63, 1.36)
Middle	0.98 (0.65, 1.48)	1.15 (0.75, 1.77)	0.84 (0.54, 1.30)
Richer	0.79 (0.50, 1.27)	0.64 (0.37, 1.10)	1.04 (0.66, 1.66)
Richest	0.67 (0.38, 1.17)	0.30 (0.15, 0.58)*	1.35 (0.75, 2.44)
Caste/ethnicity			
Brahmin/Chhetri (ref.)	1	1	1
Other Terai caste	1.45 (0.86, 2.46)	1.91 (1.07, 3.41)*	0.93 (0.56, 1.56)
Dalit	1.07 (0.76, 1.51)	1.39 (0.94, 2.05)	0.86 (0.58, 1.28)
Newar	0.84 (0.37, 1.90)	0.71 (0.31, 1.59)	0.97 (0.36, 2.62)
Janjati	0.95 (0.70, 1.28)	1.00 (0.71, 1.40)	0.94 (0.69, 1.27)
Muslim	2.47 (1.51, 4.05)*	1.15 (0.58, 2.30)	2.58 (1.47, 4.57)*
Place of residence			
Urban (ref.)	1	1	1
Rural	1.57 (1.07, 2.30)*	0.94 (0.63, 1.41)	1.83 (1.21, 2.75)*
Ecological zone			
Mountain (ref.)	1	1	1
Hill	1.27 (0.93, 1.74)	1.04 (0.71, 1.53)	1.27 (0.91, 1.75)
Terai	0.79 (0.55, 1.13)	1.24 (0.82, 1.87)	0.64 (0.42, 0.97)*

*Statistically significant confidence interval; ref: reference category.
